# Sport-Specific Balance Develops Specific Postural Skills

**DOI:** 10.1007/s40279-014-0174-x

**Published:** 2014-03-26

**Authors:** Thierry Paillard

**Affiliations:** Laboratoire Activité Physique, Performance et Santé (UPRES EA 4445), Département STAPS, Université de Pau et des Pays de l’Adour, ZA Bastillac Sud, 11 rue Morane Saulnier, 65000 Tarbes, France

I read the review by Zemková [1], entitled *Sport-Specific Balance*, with great interest. This is an important research area that may contribute to a better understanding of the postural control system under various sport performance requirements.

The main objectives of this review were to analyse the effects of various types of exercise on balance performance, and to provide new insights into the underlying physiological mechanisms that contribute to these effects. To this end, Zemková [1] investigated the postural sway response to different forms of exercise under laboratory and sport-specific conditions to examine how this effect can vary with expertise, and to provide examples of the association of impaired balance with sport performance and/or increasing risk of injury. This author deduces that athletes of different specializations have a better ability to maintain balance in specific conditions (e.g. while standing on a narrow area of support) than physically active individuals. Zemková [1] also observes differences in magnitude of balance impairment after specific exercises (rebound jumps, repeated rotations, etc.) and mainly in speed of readjustment to baseline.

All the concepts proposed by Zemková [1] are informative and enrich the two previous reviews that describe in a complementary way the relationship between sport activities and postural performance [2, 3]. Zemková [1] especially analysed sport-specific balance and the adaptation abilities of the postural system, particularly in sport-specific positions. Hence, one could expect to (also) find in this paper data describing the development of specific postural skills related to the sport practiced, particularly in sport-specific positions; however, Zemková [1] did not really deal with this. Yet, the contribution of these data would have provided further insights into the underlying physiological mechanisms related to the effects of specific exercises on balance performance. Indeed, it is known that the nature and environment of the movements involved in the practise of different sports influence postural adaptation. Each type of sport training leads to specific postural regulations [4] induced by the acquisition of specific new motor skills [5] due to the practice of the specific movements. In fact, sport training develops specific postural skills (in sport-specific positions and in particular environments) in terms of postural performance and strategy. Postural performance (or postural stability) can be characterized by the ability to minimize postural sway, and the postural strategy corresponds to the preferential involvement of short or long neuronal loops (contribution of proprioceptive information and contribution of visuo-vestibular information, respectively) in balance regulation.

In this letter, I propose to briefly illustrate these considerations with some examples. Concerning the adaptations in terms of postural performance related to the practice of the specific movements (i.e. in sport-specific positions), a study compared judoists who practiced their favorite throwing technique in either a bipedal stance or a monopedal stance [6]. This comparative postural analysis showed that on two-leg support, the judoists who practiced throwing techniques in a bipedal stance were more stable in standardized dynamic conditions than the judoists who practiced technical throwing in a monopedal stance. Conversely, on one-leg support, the judoists who practiced in a monopedal stance were more stable than those who practiced in a bipedal stance. This study suggests that the frequent repetition of particular throwing techniques during training probably develops specific postural skills (i.e. in sport-specific positions) that would be related to the refinement (improvement) of specific motor skills. Moreover, each sport stimulates particular sensory-motor chains (i.e. somatosensory, vestibular, or visual) and thus engenders preferential postural strategies for a given postural condition. Even sports with very similar motor behaviors (motor action), such as mountain biking and cycling (since both activities involve pedaling on a bicycle), generate specific sensory adaptations relative to postural control [7]. Lion et al. [7] reported that use of visual information was better in road cyclists than in mountain bikers when the postural condition was vision-dependent, while the mountain bikers made better use of proprioceptive information in postural control when the postural condition was proprioception-dependent. In fact, road cyclists practice on stable ground surfaces, which develops the relevant taking of visual information, while mountain bikers practice on unstable (irregular) ground surfaces, which facilitates the improvement of proprioceptive information [7]. Based on the results of this study, on a bicycle, the nature of the ground surface (i.e. the environment) influences the contribution of visual information and/or the contribution of proprioceptive information in postural regulation.

I agree with the paper by Zemková [7], which provides useful knowledge for designing training programs for specific sports. Nevertheless, the aspects mentioned above should not be denied by the reader and could reinforce the impact of this very interesting paper.
